# Subclinical thiamine deficiency identified by pretreatment evaluation in an esophageal cancer patient

**DOI:** 10.1038/s41430-020-00735-y

**Published:** 2020-09-07

**Authors:** Akira Yoshioka, Izumi Sato, Hideki Onishi, Mayumi Ishida

**Affiliations:** 1 Department of Clinical Oncology, Mitsubishi Kyoto Hospital, Kyoto, Japan; 2 Department of Pharmacoepidemiology, Graduate School of Medicine and Public Health, Kyoto University, Kyoto, Japan; 3 Departments of Psycho-oncology, Saitama Medical University International Medical Center, Hidaka City, Japan

**Keywords:** Metabolic disorders, Encephalopathy

## Abstract

Despite the fact that both thiamine deficiency (TD) and Wernicke encephalopathy (WE) have been observed to some degree in cancer patients, such cases of TD and/or WE reported to date have all been diagnosed after the initiation of treatment. We here report a case of TD that presented without the commonly accepted triad of WE symptoms based on a total nutritional evaluation prior to the onset of treatment for cancer. The patient was a 71-year-old man with esophageal cancer who was referred to the oncology outpatient clinic for evaluation to determine the treatment plan. Although he did not present with delirium, cerebellar signs, or ocular symptoms, TD was suspected based on a reduction in appetite lasting 2 months as thiamine stores in the body are depleted in as few as 18 days. Blood findings showed a marked decline in serum thiamine level supported, which the diagnosis of TD. This case revealed the existence of a cancer patient with subclinical TD prior to the onset of treatment for cancer. Due to the fact that TD can occur without the characteristic symptoms as in this case, we believe it is important that total nutritional evaluation of cancer patients always be considered.

## Introduction

The biologically active form of thiamine (thiamine pyrophosphate) is known to be essential to oxidative cellular metabolism [1]. Wernicke encephalopathy (WE) is a neuropsychiatric disorder resulting from severe acute or subacute thiamine deficiency (TD), and may lead to severe, irreversible brain damage (Korsakoff syndrome) if undetected or untreated [2]. This disorder is, however, often underrecognized due to its diverse range of symptoms. Characteristic symptoms include altered mental state, ataxia, and ocular symptoms; however, these are only observed in 16% of patients. On the other hand, 19% show hearing loss, bilateral visual loss and papilledema, epileptic seizures, hallucinations, stupor, behavioral disturbances and similar neurological symptoms, as well as hypotension, tachycardia, hypothermia, and other physical symptoms [1]. For this reason, the diagnosis of WE, especially in cancer patients, is often dependent on clinical suspicion [3].

TD and WE have been reported to occur not infrequently in patients with cancer who are referred for psychiatric consultation [4] and they have been observed at various stages of the cancer trajectory; i.e., postsurgery [5], during chemotherapy [6] and in the end-of-life stage [7, 8]. However, the patients reported to date were found to develop TD and/or WE after the onset of treatment for cancer.

Herein, we present a case report of an esophageal cancer patient diagnosed with TD prior to the start of cancer treatment by total nutritional assessment based on patient’s dietary intake and cancer status, despite the fact that the patient did not present with any of the commonly accepted triad of WE symptoms, In addition, he did not show any of the less common signs or symptoms or signs at the time of referral.

## Case report

A 71-year-old man diagnosed with esophageal cancer was referred for evaluation at the oncology outpatient clinic of our institution to determine a treatment plan. He had no significant medical history. He had been smoking one pack a day for 50 years. He had used to consume alcohol daily, but had been unable to drink for 3 months. He lived with his wife.

He had had epigastric pain for half a year and had experienced swallowing difficulty as well as vomiting for a month before admission. He was diagnosed with esophageal cancer at another hospital he had previously visited.

At the first visit to our institution, he showed no mental status changes or neurological abnormalities, with a blood pressure of 126/78 mmHg and pulse of 77/min. He advised staff that his appetite had been reduced to 1/3 that of normal from 2 months previously, and his weight had decreased from 58 to 50 kg over the previous 2 months.

Although there were no mental status changes, cerebellar symptoms, or abnormal eye movements, we suspected TD based on the reduced food consumption for 2 months and the rapid growth of tumors has been reported to result in excessive thiamine consumption [9].

High-performance liquid chromatography was employed to assess his serum thiamine concentration, which was found to be abnormally low (24 ng/mL; reference range: 30–70 ng/mL). The patient was, therefore, diagnosed with subclinical TD. After identification of his TD, thiamine (100 mg) was administered intravenously for 3 days. Thereafter, vitamin B1 was included in any infusions deemed necessary.

One week after his visit to the Oncology Clinic, he underwent two cycles of neoadjuvant chemotherapy using cisplatin and fluorouracil, followed 2 months by esophagectomy without complication.

Four months later, he underwent two cycles of adjuvant chemotherapy. The serum thiamine levels just before and after the adjuvant chemotherapy were 32 and 32 ng/mL, respectively.

## Discussion

We were able to detect subclinical TD at the time of cancer diagnosis and prior to the onset of treatment for cancer in this patient by total nutritional evaluation based on his dietary intake and cancer status. TD and WE have been reported in a significant number of cancer patients [2, 7, 10], with its clinical onset considered to be associated with the treatment [4]. Moreover, this patient did not exhibit the neuropsychiatric symptoms regarded as typical in patients with TD. The suspicion of TD in this patient was due to a total evaluation of nutritional status, such as decreased food intake reported by the patient, objective information such as weight loss, and the excess consumption of thiamine due to the rapid growth of the tumor. This suggests that the assessment of nutritional status in cancer patients is important at any time.

In the patient presented herein, TD was considered to have resulted from a decline in the patient’s appetite due to obstruction of the esophagus. The patient reported a ~70% decrease in appetite that had continued for a period of 2 months. His loss of appetite resulted in a loss of body weight of 8 kg over the 2 months. It is likely such a decline in appetite and body weight loss can lead to TD [11]. Of course, weight loss is a frequent symptom of esophageal cancer, but there have been no reports to date on the frequency of TD.

None of the three classical signs of WE (altered mental state, cerebellar signs, and ocular symptoms) were observed in this case. Instead, we observed a decline in appetite lasting for >2 weeks, a rapid decrease in body weight as well as the presence of a rapidly growing tumor. In patients with cancer, diagnosis of TD can be enhanced by the accurate assessment of the clinical context and a careful search for factors that could contribute to a decrease in thiamine intake [12]. Cases in which none of the classical triad of WE signs was apparent have been reported recently [4, 13, 14]. These reports include cases where serum thiamine levels were very low [15]. However, there have been no studies reporting the relationship between serum thiamine levels and the onset of symptoms. Therefore, a decline in appetite continues lasting for longer than 2 weeks and/or the rapid growth of a tumor [9, 16] should alert medical staff to the potential for TD despite the lack of definite neurological disturbances.

There is a limitation to this report. TD was suspected, but the patient was not immediately treated prophylactically by thiamine supplementation. In this, the treatment deviated from the recommended guidelines [17]. The administration of a high dose of thiamine is effective in treating TD [18, 19]. However, 20% of patients with WE do not present the classic triad of symptoms [20] and, if left untreated, this may lead to brain damage. Thus, treatment should be performed according to the guidelines.

In conclusion, this report revealed the existence of patients with subclinical TD prior to the commencement of cancer treatment and immediately after the diagnosis of the cancer. This suggests the importance of total nutritional evaluation in cancer patients from the time of diagnosis. In cases where a malignant tumor is identified by the attending physician, assessment of dietary intake should be undertaken as TD can develop not only after the onset of cancer treatment, as reported previously, but also prior to the onset of treatment. A decline in appetite lasting longer than 2 weeks and/or rapid growth in tumor size is confirmed, the potential for TD should be considered despite the lack of any changes in mental state, and the need for prophylactic thiamine treatment should be taken into consideration.

Future studies on the frequency of TD and associated factors in cancer patients prior to treatment are advised.
